# Custom bipolar electrode for bleomycin-based electrochemotherapy in patient-derived uveal melanoma *in ovo* and *ex vivo*

**DOI:** 10.3389/fcell.2026.1860536

**Published:** 2026-09-08

**Authors:** Ralitsa Anastasova, Miltiadis Fiorentzis, Azin Yousefiboroujeni, Giacomo Perazzolo Gallo, Matteo Cadossi, Sami Dalbah, Berthold Seitz, Nikolaos E. Bechrakis, Theodora Tsimpaki

**Affiliations:** 1 Department of Ophthalmology, University Hospital Essen, Essen, Germany; 2 Department of Ophthalmology, University Medical Center Schleswig-Holstein, Lübeck, Germany; 3 IGEA S.p.A., Carpi, Italy; 4 Department of Ophthalmology, Saarland University Medical Center, Homburg, Germany

**Keywords:** bleomycin, cell death, electrochemotherapy, patient-derived xenograft, uveal melanoma

## Abstract

**Introduction:**

Uveal melanoma (UM) is a rare tumor entity and the predominant primary intraocular malignancy in adulthood. Despite high local tumor control rates, metastatic disease remains frequent and systemic therapeutic options are limited. Electrochemotherapy (ECT) with bleomycin enhances intracellular drug uptake through electroporation (EP), thereby increasing targeted antitumor activity. This study investigated the effects of bleomycin-based ECT delivered using a custom bipolar electrode in UM patient-derived xenografts (PDX).

**Methods:**

UM PDX were analyzed in both *in ovo* and *ex vivo* experimental settings. Tumor grafts were treated with EP alone at voltage settings of 750 V or 1000 V, or with ECT combined with bleomycin at concentrations of 1 μg/mL or 2.5 μg/mL. Histological evaluation and immunofluorescence analyses were performed to assess tumor morphology, melanoma-associated marker expression, vascularization, proliferative activity and treatment-induced cell death pathways.

**Results:**

ECT caused pronounced tumor disruption and decreased tumor size parameters compared with untreated controls, with statistically significant reductions in tumor area following EP at 750 V alone or combined with 2.5 μg/mL bleomycin and in tumor perimeter across all groups treated with EP at 750 V *in ovo*, while changes in the *ex vivo* model were not statistically significant. Expression of melanoma-associated markers, including a melanoma marker cocktail and Sox10, decreased following treatment, with significant differences observed in multiple *in ovo* and selected *ex vivo* conditions. Analysis of cell death markers indicated involvement of multiple cell death mechanisms, with statistically significant treatment-associated changes in markers of apoptosis, pyroptosis, necrosis and necroptosis detected *in ovo*, whereas gasdermin D expression did not reach statistical significance *ex vivo*. Treatment responses differed between experimental models and EP conditions, suggesting model-dependent cellular responses.

**Discussion:**

Bleomycin-based ECT using a custom bipolar electrode induces substantial tumor damage in UM PDX *in ovo* and *ex vivo*. These findings support the potential application of bipolar electrode-based ECT for intraocular tumor treatment and provide a basis for further optimization of electrode design and translational investigation.

## 1 Introduction

Uveal melanoma (UM) arises from the malignant transformation of melanocytes within the uveal tract of the eye. UM most commonly originates in the choroid (90%) and less frequently in the ciliary body (6%) or the iris (4%) (Shields et al., 2009). The annual incidence in Europe ranges from 2 to 8 cases per million (Group et al., 2007). Epidemiological patterns vary according to the geographical location, ethnicity and gender with increased UM rates observed at higher latitudes, among individuals of Caucasian ancestry and in males (Gill et al., 2022; Shields et al., 2015; Alfaar et al., 2022). UM is associated with an unfavorable prognosis due to the high risk of metastatic dissemination and the lack of curative systemic therapies. Metastatic disease develops in up to half of affected patients, with the liver as the primary site of spread in more than 90% of cases (Group, 2001). Once metastases occur, median overall survival is less than 1 year and five-year survival rates are below 20% (Khoja et al., 2019). UM exhibits a distinct genetic profile characterized by a low mutational burden and recurrent chromosomal abnormalities, including monosomy of chromosome 3 and loss of BRCA1-associated protein 1 (BAP1), associated with aggressive disease behavior and reduced therapeutic responsiveness (Harbour et al., 2010). Management of UM focuses on local tumor control while preserving both ocular integrity and function. Radiotherapy, delivered as brachytherapy or teletherapy, is the standard treatment for most patients. Surgical approaches, including transscleral resection, endoresection or enucleation, are reserved for selected cases, such as large, recurrent or radioresistant tumors (Damato and Lecuona, 2004). Despite advances in local treatment, overall survival remains suboptimal, as the risk of metastatic progression persists. Systemic therapeutic options for UM metastases are limited, with tebentafusp, a gp100-directed T-cell receptor-based immunotherapy, demonstrating a survival benefit in a subset of patients (Nathan et al., 2021). Consequently, novel therapeutic strategies with translational potential are required.

Electrochemotherapy (ECT) combines the administration of chemotherapeutic agents, such as bleomycin, with the application of short, targeted electric pulses, referred to as electroporation (EP), to facilitate intracellular drug uptake, resulting in increased local antitumor efficacy while minimizing systemic toxicity (Campana et al., 2019). Due to its localized mechanism of action and precise pulse delivery, ECT is well suited for anatomically sensitive or surgically challenging sites (Linnert et al., 2012). While ECT has shown efficacy across multiple tumor types, its implementation in UM has been explored predominantly in preclinical settings. Studies using UM cell-based systems and patient-derived xenografts (PDX) implanted on the chorioallantoic membrane (CAM) have demonstrated the feasibility and antitumor activity of bleomycin-based ECT with plate electrodes *in ovo* (Tsimpaki et al., 2024; Anastasova et al., 2024). However, optimization of electrode design for intraocular use remains largely unexplored. To address this limitation, a custom bipolar electrode was designed to enable intratumoral insertion and effective ablation of larger tumor masses, supporting its potential intraocular application. The aim of this study was to evaluate the impact of a bipolar electrode configuration on tumor cell death after ECT with bleomycin in a UM PDX model using *in ovo* and *ex vivo* experimental systems, assessed by histological and immunofluorescence analyses.

## 2 Materials and methods

### 2.1 Patient cohort and tumor sample collection

Between July 2023 and January 2025, twelve patients with UM were included in this study, eight females and four males, with a mean age at enucleation of 67 ± 14 years. Chromosome 3 analysis revealed disomy in six cases and monosomy in two, while no chromosomal data were obtained for the remaining four patients due to individual preference. Loss of BAP1 expression was identified in four tumor samples. Histopathological evaluation demonstrated predominantly spindle cell morphology, with one epithelioid and one mixed-cell tumor. This study was approved by the ethics committee of the Faculty of Medicine of University of Duisburg-Essen (No. 21-9959-BO) and conducted in accordance with the Declaration of Helsinki. Tumor samples were collected from enucleated eye globes and transferred into medium (RPMI 1640 medium, Gibco, Thermo Fisher Scientific, Waltham, MA, United States). Specimens were stored on ice and processed immediately using *in ovo* or *ex vivo* experimental setups. Fresh tumor tissues were sectioned into fragments, with 67 samples allocated to *in ovo* experiments and 33 to *ex vivo*.

### 2.2 Bipolar electrode configuration and electrochemotherapy settings

**FIGURE 1:**
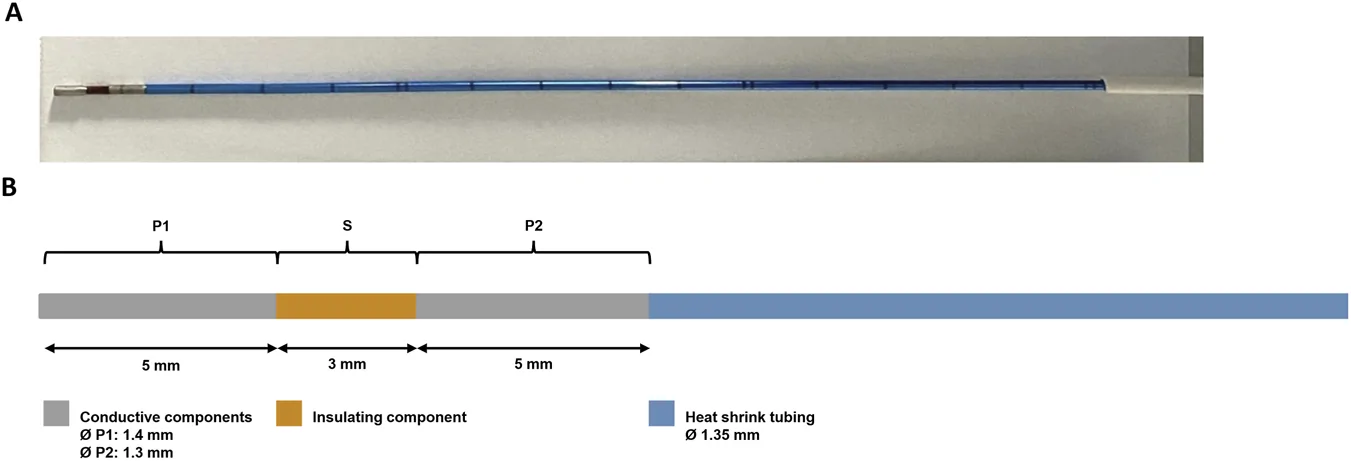
Custom bipolar electrode. **(A)** Representative image of the custom bipolar electrode used in this study. **(B)** Schematic representation of the custom bipolar electrode composed of a distal conductive pole (P1), an insulated spacer (S), a proximal conductive pole (P2) and a heat shrink tubing. The illustration is not drawn to scale.

A custom-designed minimally invasive bipolar electrode (IGEA S. p.A., Carpi, MO, Italy) with a coaxial geometry was developed for intratumoral ECT (Figure 1A). The electrode integrated both the anode and cathode within the same needle-like structure, enabling localized electric field generation through intratumoral insertion. The electrode consisted of concentric conductive and insulating layers (Figure 1B). An inner AISI 304 stainless steel needle measuring 5 mm in length and 1.4 mm in diameter and terminating in a blunt tip formed the distal conductive pole (P1). A 3 mm hollow concentric tube then served as an insulated spacer (S). The proximal conductive pole (P2) of 5 mm in length and 1.3 mm in diameter also manufactured from AISI 304 stainless steel was positioned next, while an external insulating layer composed of heat shrink tubing with a diameter of 1.35 mm provided electrical insulation along the outer electrode sheath. The concentric tube design minimized dimensions while maintaining functional separation of conductive elements.

ECT was performed by intratumoral administration of bleomycin (bleomycin sulphate from *Streptomyces verticillus*, Sigma-Aldrich, St. Louis, MO, United States), followed by delivery of electric pulses using a commercially available pulse generator (Genedrive, IGEA S. p.A., Carpi, MO, Italy). Experiments included untreated controls, EP at 750 V or 1000 V alone and ECT with bleomycin at 1 μg/mL or 2.5 μg/mL. The selected EP settings and bleomycin concentrations were based on previous studies evaluating EP and bleomycin-based ECT, which demonstrated effective treatment responses across different experimental models, including two-dimensional (2D) cell cultures, three-dimensional (3D) spheroids and CAM-based systems (Fiorentzis et al., 2018; Fiorentzis et al., 2019; Fiorentzis et al., 2020; Tsimpaki et al., 2024; Anastasova et al., 2024). In both *in ovo* and *ex vivo* models, eight square-wave electric pulses with 100 µs duration at 5 Hz were applied. Pulse parameters remained constant, while applied voltage was varied in EP and bleomycin concentration in ECT experiments. Due to the coaxial bipolar configuration, the resulting electric field distribution is non-uniform and cannot be described by a single defined electric field strength.

### 2.3 *In ovo* treatment

**FIGURE 2:**
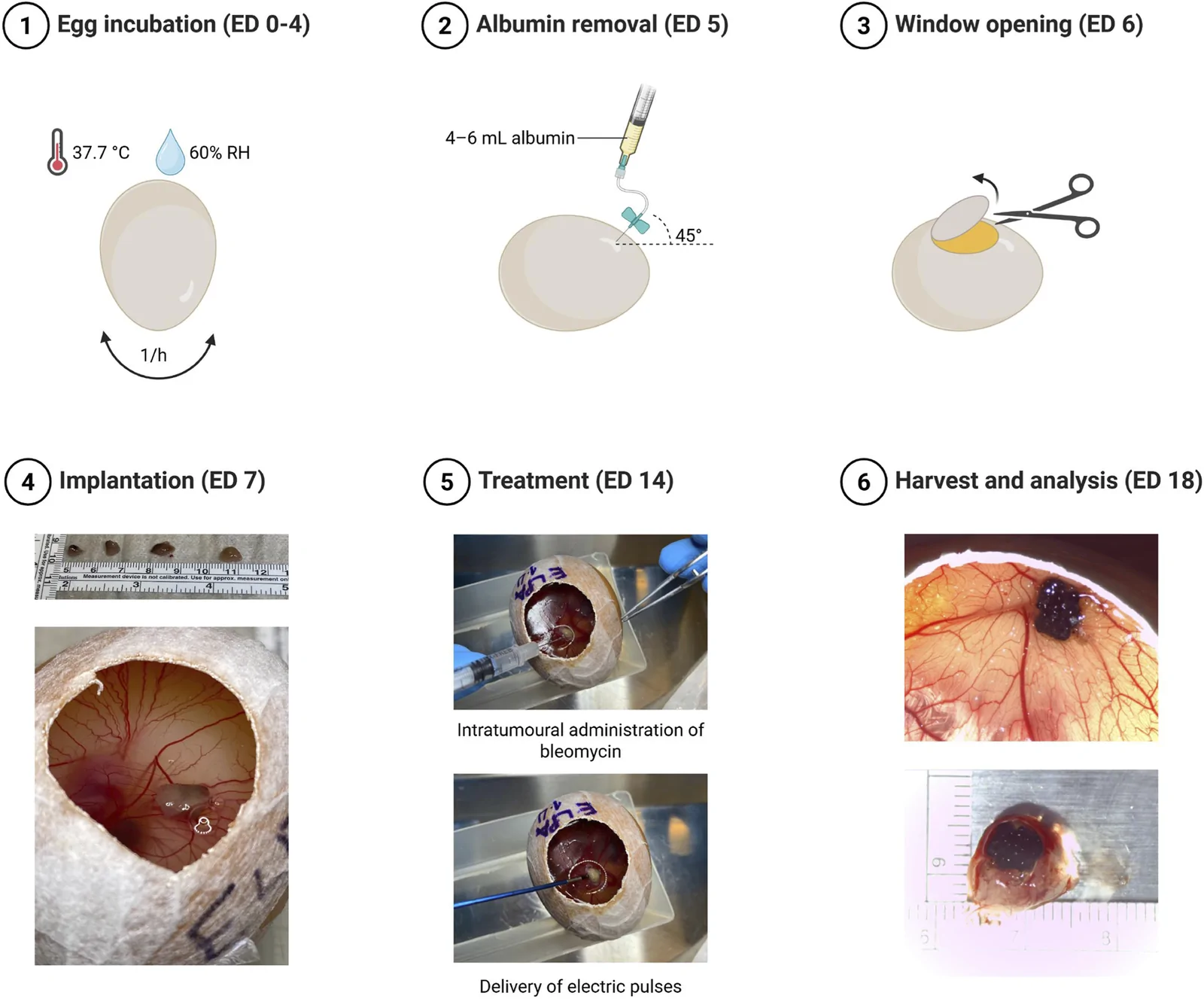
Schematic overview of the *in ovo* experimental timeline. Fertilized chicken eggs were incubated from embryonic development day 0 (ED 0) to ED 4. Albumin was removed on ED 5, followed by opening of a window in the eggshell on ED 6. Uveal melanoma (UM) patient-derived xenograft (PDX) fragments were implanted onto the chorioallantoic membrane (CAM) on ED 7. Bleomycin-based electrochemotherapy (ECT) treatment was applied on ED 14. Tumor samples were harvested on ED 18 and prepared for downstream analyses.

On embryonic development day 0 (ED 0), fertilized chicken eggs (Lohmann Deutschland, Ankum, Germany) were disinfected with 50% ethanol and placed upright in a breeding-machine incubator (Bruja 3,000 digital, Siepmann, Herdecke, Germany) at 37.7 °C and 60% relative humidity (RH) under automated rotation once per hour (Figure 2). On ED 5, rotation was terminated and 4–6 mL of albumin was aseptically aspirated using a sterile syringe (BD Discardit II syringe, Becton, Dickinson and Company, Franklin Lakes, NJ, United States) and a sterile butterfly needle (Safety-Multifly needle, Sarstedt, Nümbrecht, Germany) to detach and lower the CAM. Surgical tape (Micropore surgical tape, 3M, Saint Paul, MN, United States) was used to seal the puncture site. On ED 6, a small aseptic window was created in the eggshell to expose the CAM. The opening was covered with sterile parafilm and eggs were returned to the incubator until tumor implantation. Before engraftment on ED 7, a gentle laceration of the CAM was performed at a visible vascular branching point using a spoon-shaped curette. Matrigel (Matrigel matrix, Corning, NY, United States) was applied to the lacerated area and fresh tumor fragments obtained after enucleation were positioned onto the Matrigel droplet. The eggshell windows were sealed with sterile parafilm and incubation continued until bleomycin-based ECT treatment on ED 14, as described above. Each experimental condition included three to seven independent biological replicates. Embryo viability and tumor growth were monitored daily. In total, 67 fertilized eggs were implanted, of which 52 were subject to the treatment conditions. On ED 18, all 39 viable tumor grafts were harvested, corresponding to an egg survival rate of 58%. Tumor imaging was performed using a digital microscope camera (Leica IC80 HD, Leica Microsystems, Wetzlar, Germany) mounted on a stereomicroscope (Leica M80, Leica Microsystems, Wetzlar, Germany). Tumor samples were fixed overnight in 4% formaldehyde (ROTI Histofix, Carl Roth, Karlsruhe, Germany) and then stored in 1% phosphate-buffered saline (PBS, Sigma-Aldrich, St. Louis, MO, United States) until further processing. Embryos were sacrificed by decapitation and the lower CAM was inspected for secondary tumor foci and pigmentation.

### 2.4 *Ex vivo* treatment

Fresh *ex vivo* tumor samples obtained after enucleation were treated immediately after collection, as described above. Each experimental condition included four to nine independent biological replicates. Prior to fixation, photographic documentation of tumor samples was acquired.

### 2.5 Tumor sample processing

Following fixation, tumor samples were dehydrated in graded alcohols, cleared in xylene and embedded in paraffin. Paraffin blocks were sectioned at a thickness of 4 μm, mounted on glass slides and dried overnight at room temperature. Prior to staining, sections were deparaffinized.

### 2.6 Histology

Hematoxylin and eosin (H&E) staining enabled the evaluation of histological features of tumor samples, such as morphological characteristics, CAM integration, growth patterns and migratory behavior. An automated stitching function was used for brightfield imaging with a fluorescence microscope (BZ-X810, Keyence, Osaka, Japan). Histological images were analyzed in Fiji ImageJ (National Institutes of Health, Bethesda, MD, United States). Tumor size parameters, including area, perimeter and Feret’s diameter were quantified for each treatment condition.

### 2.7 Immunofluorescence

Immunofluorescence staining was performed to assess tumor cell identity, vascularization, proliferation and multiple cell death pathways, including apoptosis, pyroptosis, necrosis and necroptosis. After antigen retrieval and blocking, sections were incubated with primary antibodies overnight at 4 °C with melanoma marker cocktail comprising HMB45, two clones of melan-A and tyrosinase (1:200, Abcam, Cambridge, United Kingdom), Sox10 (1:200, Santa Cruz Biotechnology, Dallas, TX, United States), CD31 (1:50, Invitrogen, Waltham, MA, United States), Ki-67 (1:150, Cell Signaling Technologies, Danvers, MA, United States), cleaved caspase-3 (1:400, Cell Signaling Technologies, Danvers, MA, United States), gasdermin D (1:400, Cell Signaling Technologies, Danvers, MA, United States), HMGB1 (1:400, Abcam, Cambridge, United Kingdom) and RIP3 (1:100, Cell Signaling Technologies, Danvers, MA, United States). Following washing, Alexa Fluor 488- or Alexa Fluor 594-conjugated secondary antibodies (1:400, Invitrogen, Waltham, MA, United States) were applied for 30 min at room temperature protected from light. Nuclei were counterstained with DAPI (1:1,000, Carl Roth, Karlsruhe, Germany). Samples were prepared with liquid mountant (ProLong gold antifade mountant, Invitrogen, Waltham, MA, United States) and coverslip for imaging using a fluorescence microscope (Olympus BX51, Olympus Corporation, Tokyo, Japan). Quantitative analysis was conducted in Fiji ImageJ. Positive marker expression was determined by counting positive cells within a standardized area in three representative regions per section and mean values were calculated.

### 2.8 Statistical analysis

Experimental data were analyzed with GraphPad Prism (GraphPad Software, San Diego, CA, United States). Two-way ANOVA was used for group comparisons. Statistical significance was defined as p < 0.05 and was denoted as *p ≤ 0.05, **p ≤ 0.01, ***p ≤ 0.005, and ****p ≤ 0.001.

## 3 Results

### 3.1 Histological evaluation of patient-derived xenografts

**FIGURE 3:**
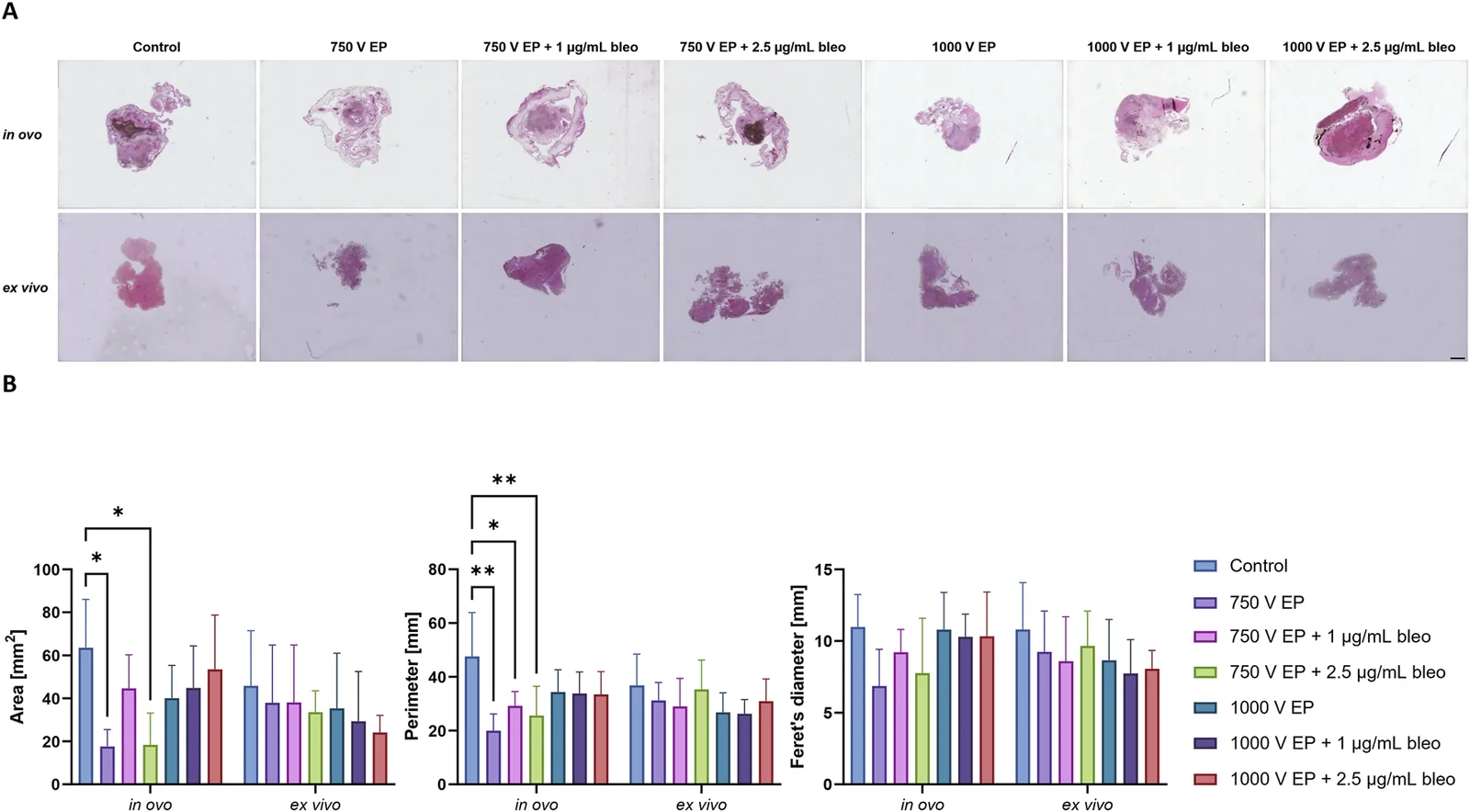
Histological analysis of patient-derived xenografts (PDX). **(A)** Representative histological images showing hematoxylin and eosin (H&E) staining of *in ovo* and *ex vivo* tumor grafts after electroporation (EP) alone or after electrochemotherapy (ECT) with bleomycin compared with untreated controls. Scale bar, 1 mm. **(B)** Quantification of tumor area, perimeter and Feret’s diameter in *in ovo* and *ex vivo* tumor grafts after EP alone or after ECT with bleomycin compared with untreated controls. Data are presented as mean ± SD. Each experimental condition included 3-7 independent biological replicates *in ovo* and 4-9 independent biological replicates *ex vivo*. Statistical analysis was performed using two-way ANOVA. Statistical significance levels are indicated with *p ≤ 0.05, **p ≤ 0.01.

Histological examination after H&E staining revealed treatment-dependent morphological alterations in the *in ovo* model. Untreated tumor grafts displayed compact cellular organization with focal central necrotic areas and defined integration into the CAM (Figure 3A). Following treatment, tumor morphology appeared disorganized with reduced cellular cohesion and irregular tumor margins. Treated tumors exhibited more extensive and heterogeneously necrotic regions than controls. Quantitative analysis demonstrated reductions in tumor area, perimeter and Feret’s diameter following treatment relative to controls (Figure 3B). A significant decrease in tumor area was observed after EP at 750 V alone or in combination with 2.5 μg/mL bleomycin. A similar pattern was evident for tumor perimeter, with significant reductions across all 750 V EP conditions. Feret’s diameter was decreased across all 750 V EP groups without reaching statistical significance. In the *ex vivo* setting, tumor grafts exhibited fewer necrotic regions among all conditions and a more dispersed tissue structure following treatment (Figure 3A). All treated samples were associated with non-significant reductions in tumor area, perimeter and Feret’s diameter (Figure 3B).

### 3.2 Immunofluorescence-based characterization of patient-derived xenografts

#### 3.2.1 Tumor identity

**FIGURE 4:**
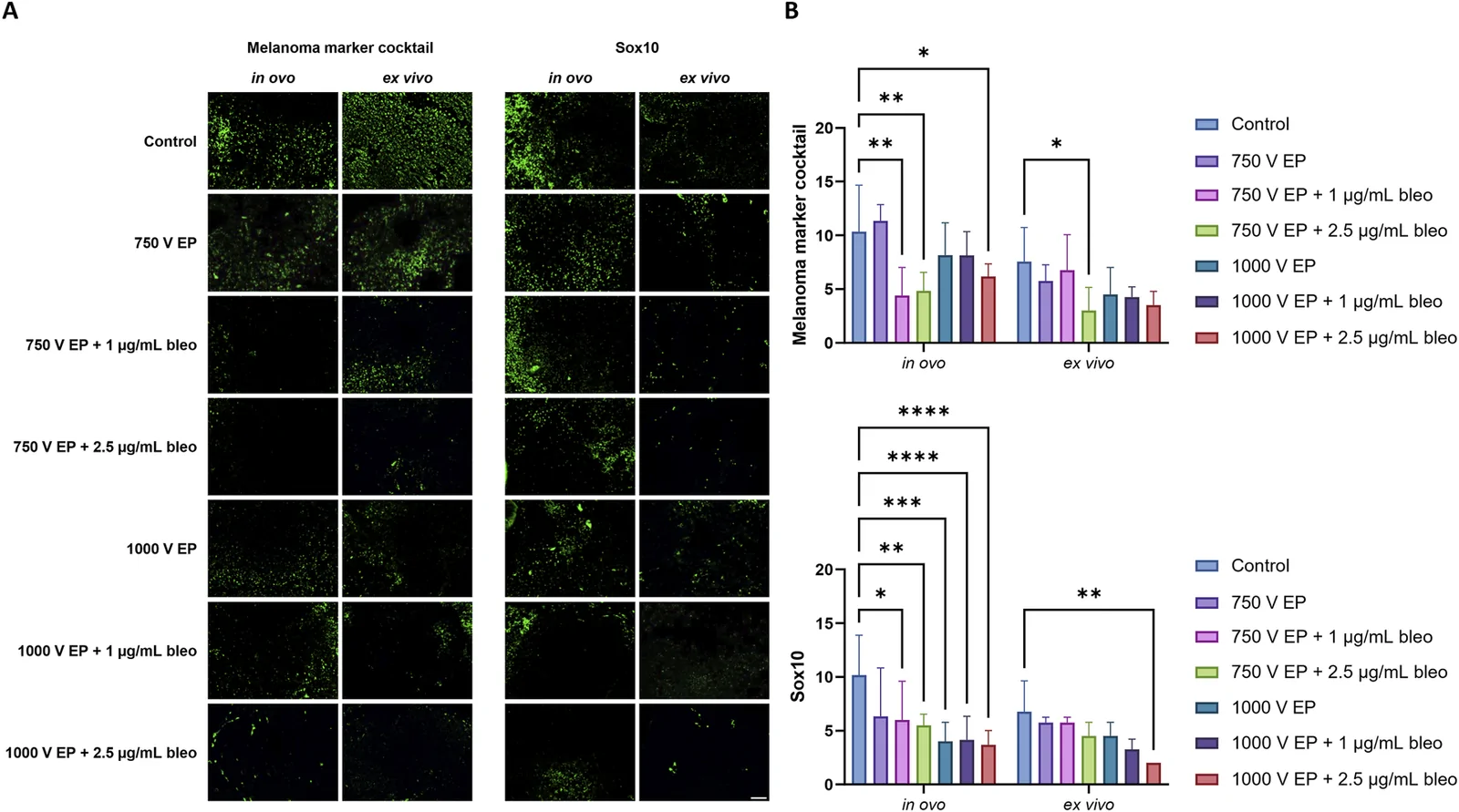
Immunofluorescence analysis of tumor identity markers in patient-derived xenografts (PDX). **(A)** Representative immunofluorescence images showing melanoma marker cocktail and Sox10 expression in *in ovo* and *ex vivo* tumor grafts after electroporation (EP) alone or after electrochemotherapy (ECT) with bleomycin compared with untreated controls. Scale bar, 100 µm. **(B)** Quantification of immunofluorescence signals expressed as positive cell counts per calculated area in *in ovo* and *ex vivo* tumor grafts after EP alone or after ECT with bleomycin compared with untreated controls. Data are presented as mean ± SD. Each experimental condition included 3-7 independent biological replicates *in ovo* and 4-9 independent biological replicates *ex vivo*. Statistical analysis was performed using two-way ANOVA. Statistical significance levels are indicated with *p ≤ 0.05, **p ≤ 0.01, ***p ≤ 0.005, ****p ≤ 0.001.

The preservation of tumor identity was investigated using immunofluorescent detection of melanoma marker cocktail and Sox10. In the *in ovo* system, untreated tumor grafts showed strong and homogeneous staining for both markers. Treatment reduced melanoma marker positivity, with the most pronounced effect observed following bleomycin-based ECT (Figure 4A). While EP at 750 V alone maintained expression levels comparable to controls, addition of bleomycin caused a significant decrease in melanoma marker positive cells, independent of voltage (Figure 4B). At 1000 V EP, marker abundance was lower than in controls, with further significant decline with increasing bleomycin concentration. Sox10 followed a similar trend, with significant reduction across all treated *in ovo* groups, except for EP at 750 V alone, where the decrease was non-significant. In the *ex vivo* setting, untreated tumor samples likewise exhibited high expression of melanoma marker and Sox10, which decreased after treatment (Figure 4A). Significant decline in melanoma marker positive cells was detected following EP at 750 V combined with 2.5 μg/mL bleomycin, whereas EP at 1000 V induced a dose-dependent decrease that did not reach statistical significance (Figure 4B). Sox10 positivity was significantly reduced after treatment with EP at 1000 V together with 2.5 μg/mL bleomycin, while other conditions showed non-significant decreases.

#### 3.2.2 Tumor vascularization and proliferation

**FIGURE 5:**
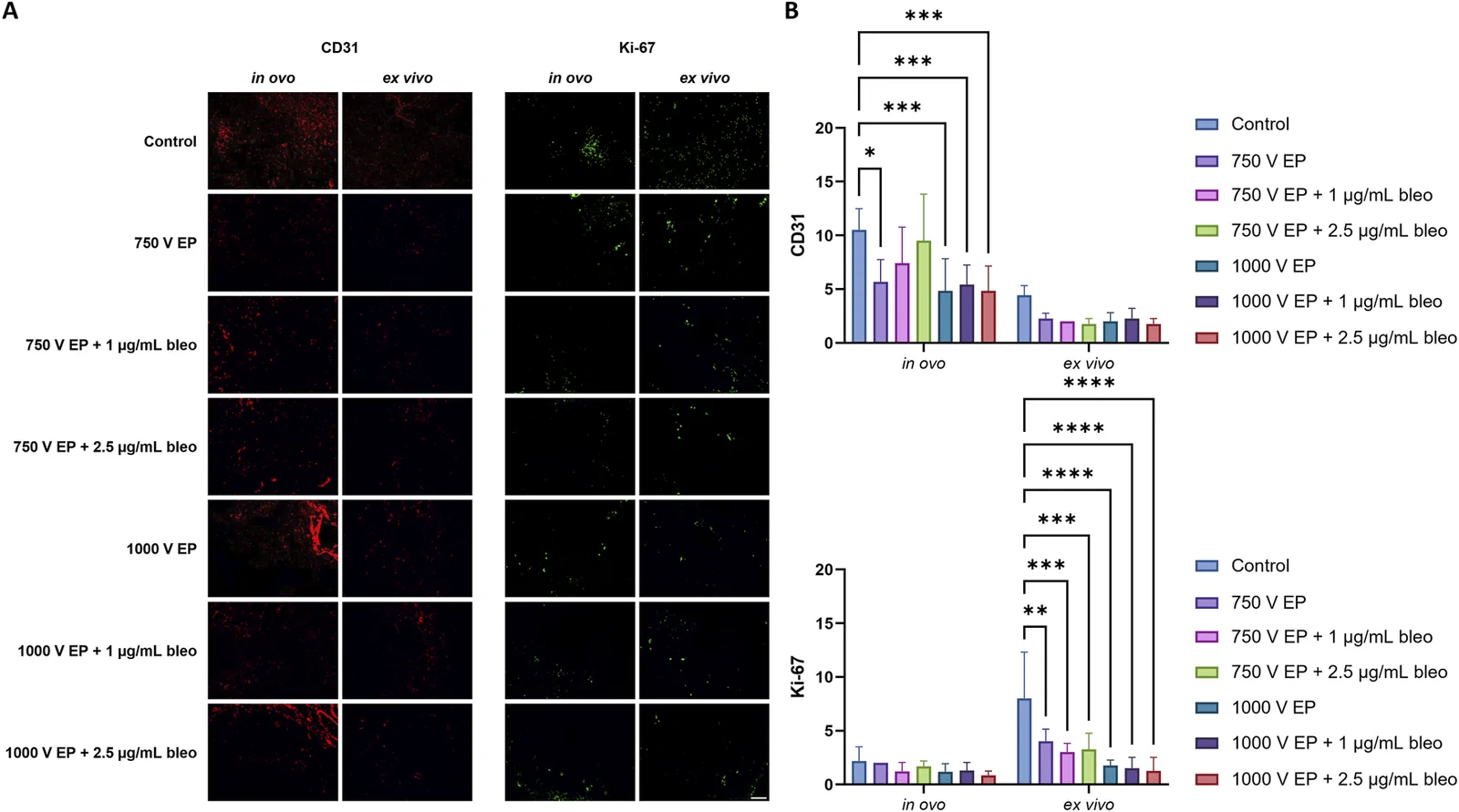
Immunofluorescence analysis of tumor vascularization and proliferation markers in patient-derived xenografts (PDX). **(A)** Representative immunofluorescence images showing CD31 and Ki-67 expression in *in ovo* and *ex vivo* tumor grafts after electroporation (EP) alone or after electrochemotherapy (ECT) with bleomycin compared with untreated controls. Scale bar, 100 µm. **(B)** Quantification of immunofluorescence signals expressed as positive cell counts per calculated area in *in ovo* and *ex vivo* tumor grafts after EP alone or after ECT with bleomycin compared with untreated controls. Data are presented as mean ± SD. Each experimental condition included 3-7 independent biological replicates *in ovo* and 4-9 independent biological replicates *ex vivo*. Statistical analysis was performed using two-way ANOVA. Statistical significance levels are indicated with *p ≤ 0.05, **p ≤ 0.01, ***p ≤ 0.005, ****p ≤ 0.001.

Changes in tumor vascularization and proliferation activity were examined by immunofluorescent staining for CD31 and Ki-67, respectively. *In ovo*, untreated samples were highly vascularized, whereas CD31 expression declined after treatment, particularly following 750 V EP alone and among all EP conditions at 1000 V (Figure 5A). Despite reduced intratumoral vascularization, vascular sprouting of CAM-derived vessels into the tumor graft confirmed successful engraftment. Quantitative analysis revealed significant reduction in CD31 positive cells after EP at 750 V alone and in all 1000 V EP groups (Figure 5B). In contrast, *in ovo* Ki-67 staining remained uniformly low across all groups, including controls, with no significant differences detected. *Ex vivo*, CD31 staining showed minimal variation between untreated and treated tumor grafts with no significant changes. Proliferative activity was highest in the control group and decreased following treatment under all conditions, with significant reduction in Ki-67 positive cells.

#### 3.2.3 Apoptosis

**FIGURE 6:**
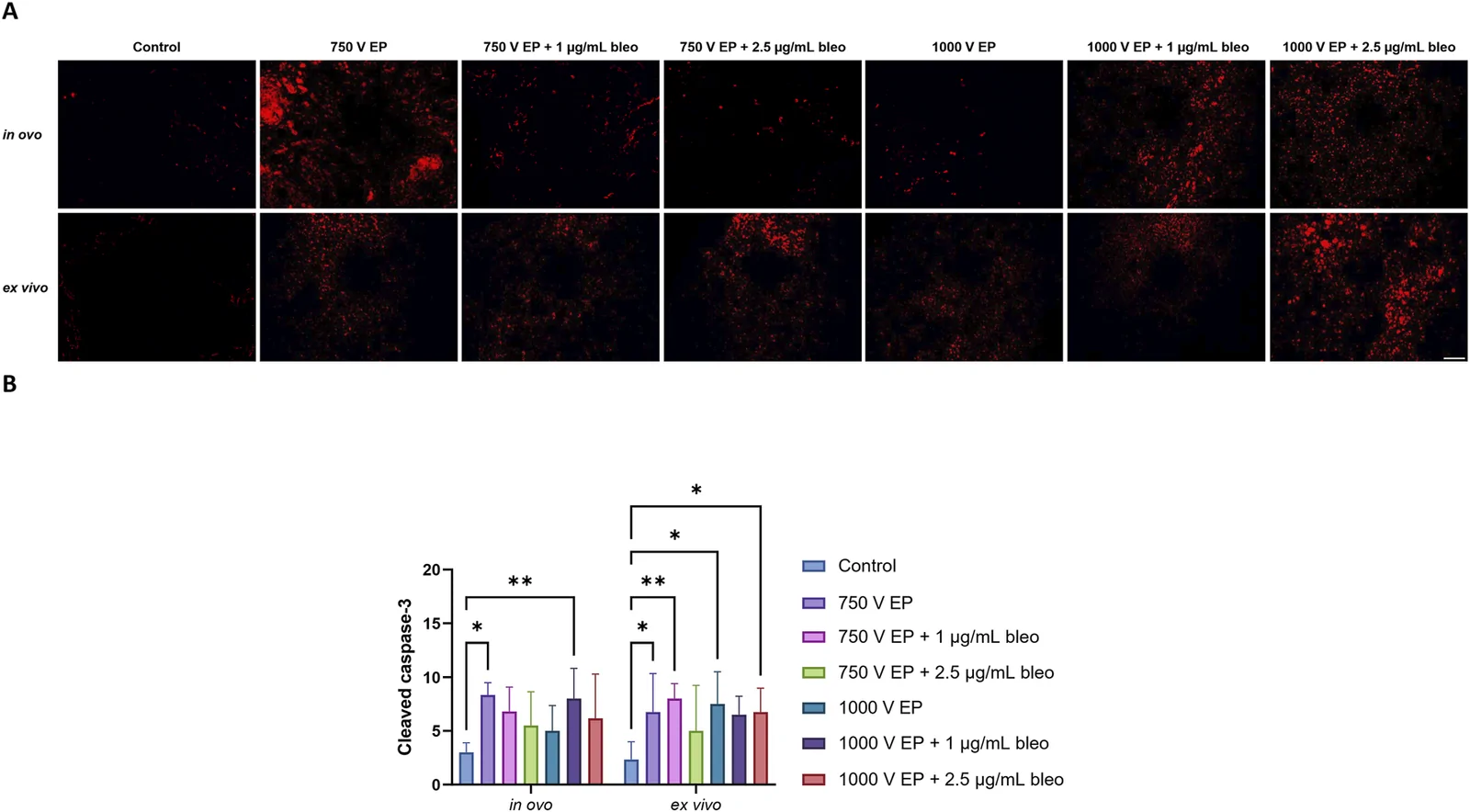
Immunofluorescence analysis of treatment-induced apoptosis in patient-derived xenografts (PDX). **(A)** Representative immunofluorescence images showing cleaved caspase-3 expression in *in ovo* and *ex vivo* tumor grafts after electroporation (EP) alone or after electrochemotherapy (ECT) with bleomycin compared with untreated controls. Scale bar, 100 µm. **(B)** Quantification of immunofluorescence signals expressed as positive cell counts per calculated area in *in ovo* and *ex vivo* tumor grafts after EP alone or after ECT with bleomycin compared with untreated controls. Data are presented as mean ± SD. Each experimental condition included 3-7 independent biological replicates *in ovo* and 4-9 independent biological replicates *ex vivo*. Statistical analysis was performed using two-way ANOVA. Statistical significance levels are indicated with *p ≤ 0.05, **p ≤ 0.01.

Apoptotic responses were assessed by immunofluorescent detection of cleaved caspase-3. In the *in ovo* model, signal intensity was increased in tumor grafts treated with EP at 750 V alone and with EP at 1000 V in combination with bleomycin at either concentration compared with untreated controls (Figure 6A). Quantification revealed a significant rise in cleaved caspase-3 positive cells following EP at 750 V alone and EP at 1000 V together with bleomycin at a concentration of 1 μg/mL in comparison to the control group (Figure 6B). In the *ex vivo* tumor samples, all treatment conditions produced a moderate increase in cleaved caspase-3 fluorescence, with a more prominent and homogeneously distributed signal observed at the higher EP setting with the higher bleomycin concentration (Figure 6A). Both EP only conditions resulted in significantly elevated apoptotic marker positivity relative to the controls (Figure 6B). Additionally, a significant increase was detected after combined treatment with 750 V EP and 1 μg/mL bleomycin, as well as with 1000 V EP and 2.5 μg/mL bleomycin.

#### 3.2.4 Pyroptosis

**FIGURE 7:**
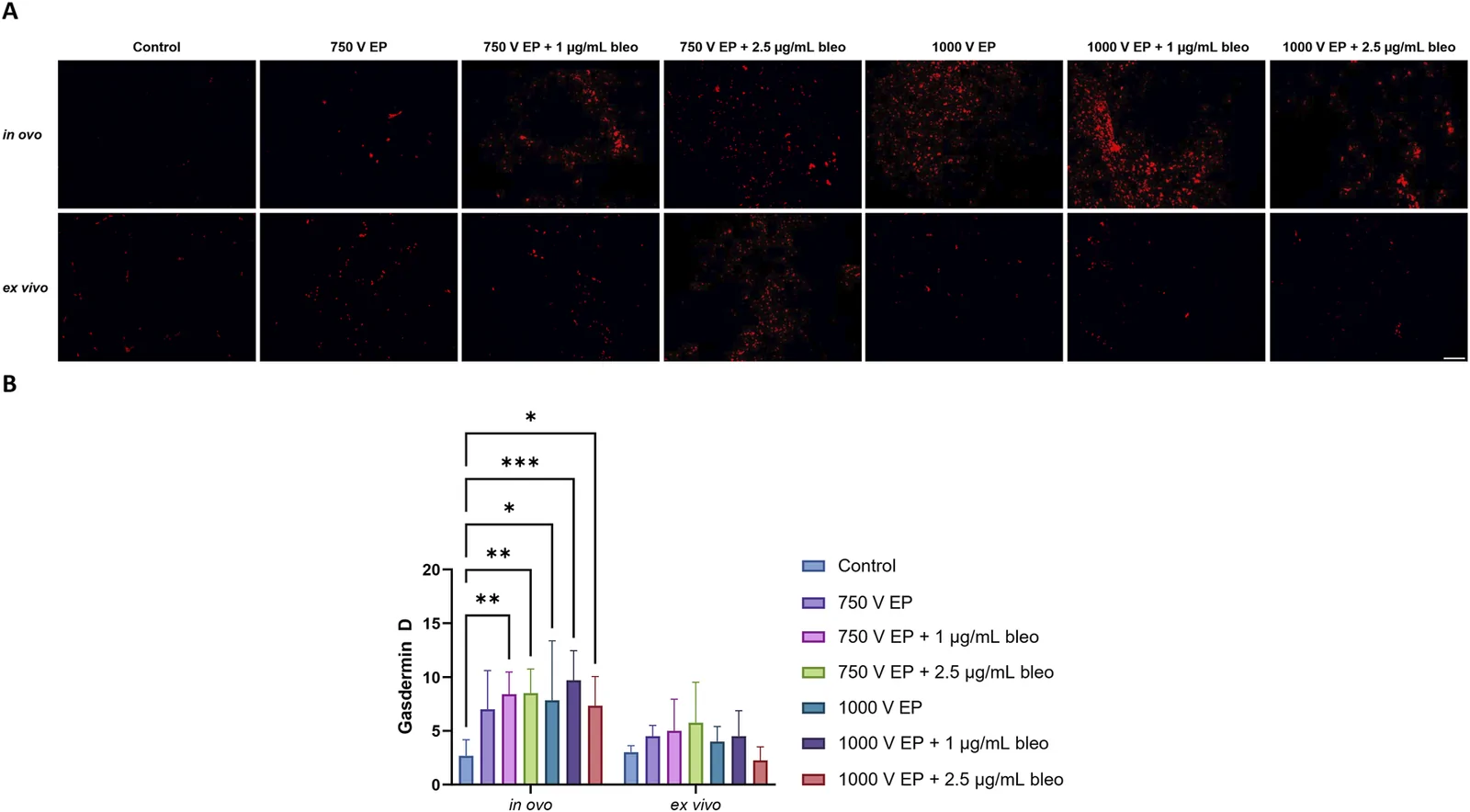
Immunofluorescence analysis of treatment-induced pyroptosis in patient-derived xenografts (PDX). **(A)** Representative immunofluorescence images showing gasdermin D expression in *in ovo* and *ex vivo* tumor grafts after electroporation (EP) alone or after electrochemotherapy (ECT) with bleomycin compared with untreated controls. Scale bar, 100 µm. **(B)** Quantification of immunofluorescence signals expressed as positive cell counts per calculated area in *in ovo* and *ex vivo* tumor grafts after EP alone or after ECT with bleomycin compared with untreated controls. Data are presented as mean ± SD. Each experimental condition included 3-7 independent biological replicates *in ovo* and 4-9 independent biological replicates *ex vivo*. Statistical analysis was performed using two-way ANOVA. Statistical significance levels are indicated with *p ≤ 0.05, **p ≤ 0.01, ***p ≤ 0.005.

Pyroptosis-associated changes were evaluated by gasdermin D expression. In the *in ovo* setting, gasdermin D positivity was present across all treated samples, with weaker staining following EP at 750 V alone (Figure 7A). Consistently, a significant pyroptotic response was evident in all treatment groups relative to the controls, except for EP at 750 V alone, which exhibited a marked but non-significant increase (Figure 7B). In *ex vivo* tumor samples, gasdermin D expression was sparse, with a more diffuse staining pattern after treatment with 750 V EP administered with 2.5 μg/mL bleomycin (Figure 7A). Although this correlated with an increase in gasdermin D positive cells, no significant differences were identified compared with untreated tumor grafts (Figure 7B).

#### 3.2.5 Necrosis

**FIGURE 8:**
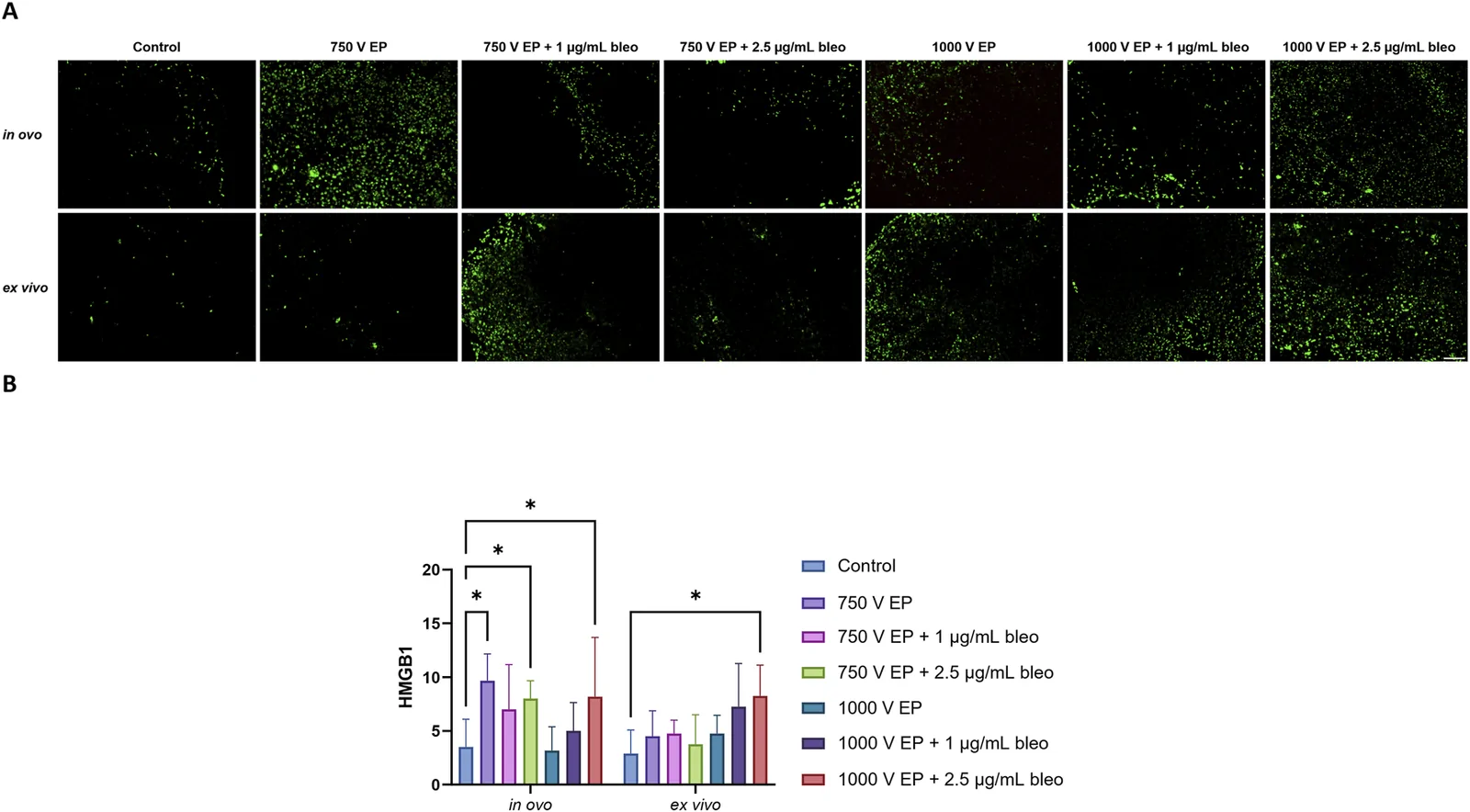
Immunofluorescence analysis of treatment-induced necrosis in patient-derived xenografts (PDX). **(A)** Representative immunofluorescence images showing HMGB1 expression in *in ovo* and *ex vivo* tumor grafts after electroporation (EP) alone or after electrochemotherapy (ECT) with bleomycin compared with untreated controls. Scale bar, 100 µm. **(B)** Quantification of immunofluorescence signals expressed as positive cell counts per calculated area in *in ovo* and *ex vivo* tumor grafts after EP alone or after ECT with bleomycin compared with untreated controls. Data are presented as mean ± SD. Each experimental condition included 3-7 independent biological replicates *in ovo* and 4-9 independent biological replicates *ex vivo*. Statistical analysis was performed using two-way ANOVA. Statistical significance levels are indicated with *p ≤ 0.05.

Immunofluorescence analysis of HMGB1 was used to characterize necrotic cell death. In the *in ovo* system, intense HMGB1 staining was apparent after EP at 750 V alone and remained prominent across all other EP-treated conditions, whereas the control group appeared only weakly positive (Figure 8A). Treatment with 750 V EP alone and either 750 V or 1000 V EP applied with 2.5 μg/mL bleomycin resulted in significantly increased necrotic marker levels (Figure 8B). *Ex vivo*, HMGB1 positivity was limited in untreated samples and in tumor grafts treated with EP at 750 V alone or EP at 750 V in combination with 2.5 μg/mL bleomycin (Figure 8A). In contrast, enhanced HMGB1 expression was predominantly localized at the tumor margins following treatment with 750 V EP administered with 1 μg/mL bleomycin, 1000 V EP alone and 1000 V EP together with 1 μg/mL bleomycin. A strong and homogeneous HMGB1 staining pattern observed after EP at 1000 V with bleomycin at a concentration of 2.5 μg/mL confirmed a significant difference compared with the controls (Figure 8B).

#### 3.2.6 Necroptosis

**FIGURE 9:**
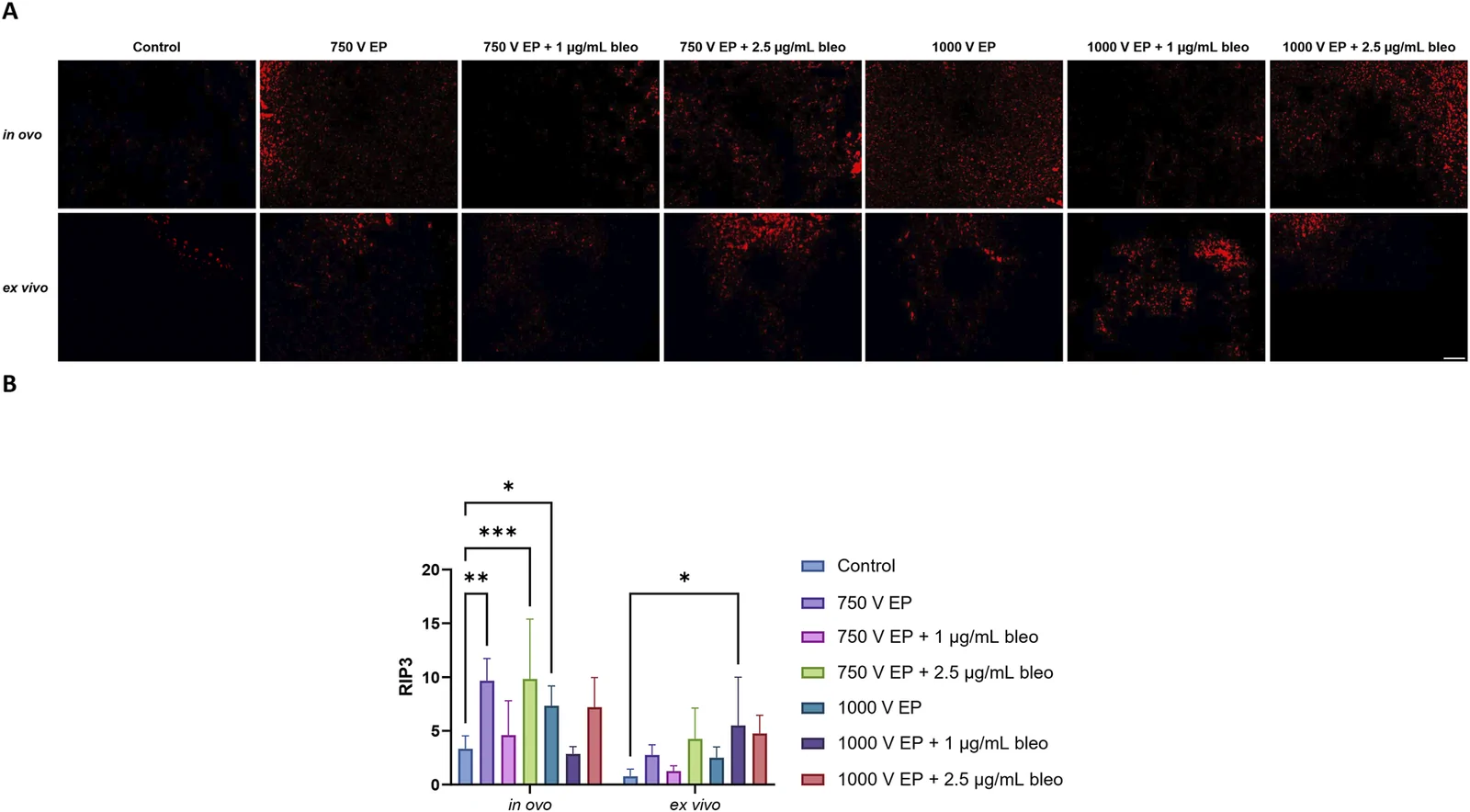
Immunofluorescence analysis of treatment-induced necroptosis in patient-derived xenografts (PDX). **(A)** Representative immunofluorescence images showing RIP3 expression in *in ovo* and *ex vivo* tumor grafts after electroporation (EP) alone or after electrochemotherapy (ECT) with bleomycin compared with untreated controls. Scale bar, 100 µm. **(B)** Quantification of immunofluorescence signals expressed as positive cell counts per calculated area in *in ovo* and *ex vivo* tumor grafts after EP alone or after ECT with bleomycin compared with untreated controls. Data are presented as mean ± SD. Each experimental condition included 3-7 independent biological replicates *in ovo* and 4-9 independent biological replicates *ex vivo*. Statistical analysis was performed using two-way ANOVA. Statistical significance levels are indicated with *p ≤ 0.05, **p ≤ 0.01, ***p ≤ 0.005.

RIP3 served as a marker for the immunofluorescence-based assessment of necroptosis. In the *in ovo* model, a strong RIP3 signal was detected following both EP only conditions, with further enhancement at higher bleomycin concentration relative to controls (Figure 9A). Accordingly, a significant increase in RIP3 positive cells was observed after treatment with EP at 750 V or 1000 V alone and after 750 V EP together with 2.5 μg/mL bleomycin (Figure 9B). Although not reaching statistical significance, EP at 1000 V in combination with the higher bleomycin concentration showed a pronounced rise in RIP3 expression. In *ex vivo* tumor grafts, RIP3 staining was weak and dispersed, with increased signal intensity at 750 V EP combined with 2.5 μg/mL bleomycin and with 1000 V EP administered with 1 μg/mL bleomycin (Figure 9A). Correspondingly, EP at 1000 V together with 1 μg/mL bleomycin resulted in significantly elevated RIP3 positivity (Figure 9B).

## 4 Discussion

This study demonstrates that bleomycin-based ECT using a custom bipolar electrode induces tumor disruption and multimodal cell death in UM PDX. Treatment reduced tumor size parameters and melanoma-related marker expression, while indicating the involvement of multiple cell death pathways. The therapeutic effect was more pronounced in the *in ovo* setting than in the *ex vivo* tumor samples, indicating a microenvironment-dependent response. These findings support the biological activity and feasibility of an intratumoral bipolar electrode configuration for ECT in UM.

Although effective treatment strategies for primary UM are available, metastatic dissemination still occurs in approximately 40%–50% of UM patients despite adequate local tumor control (Rantala et al., 2021). The liver represents the predominant site of metastatic involvement, whereas extrahepatic spread is associated with a widespread systemic disease and poor prognosis (Garg et al., 2022). Systemic therapeutic options for metastatic UM remain limited, as chemotherapy demonstrates minimal efficacy and immunotherapy only a modest benefit (Pons et al., 2011; Algazi et al., 2016). Tebentafusp, the first-in-class systemic agent to show a survival advantage, is restricted to patients expressing HLA-A*02:01 (Nathan et al., 2021). Consequently, many patients still lack effective systemic or adjuvant treatment options.

ECT enhances the cytotoxicity of chemotherapeutic agents, such as bleomycin, through reversible EP (Probst et al., 2018). Transient membrane permeabilization enables intracellular bleomycin accumulation, inducing DNA damage and tumor cell death while limiting systemic toxicity (Hecht, 2000). ECT has demonstrated clinical efficacy across a broad range of tumor types (Heller et al., 1998; Strojan et al., 2021; Campana et al., 2014; Bonferoni et al., 2021). In ophthalmology, ECT has been applied to periocular and ocular surface malignancies, with favorable tumor responses and preservation of ocular function (Kis et al., 2019; Salwa et al., 2014). More recent clinical evidence has demonstrated high response rates in locally advanced or recurrent basal cell carcinoma of the eyelid and periocular region (Vass et al., 2025). Veterinary reports have further described successful bleomycin-based ECT of ocular malignancies, including squamous cell carcinoma of the equine ocular surface and feline third eyelid, with sustained local tumor control observed in individual cases (Larsen et al., 2025; Peña and Ferrer, 2026). ECT has been applied to malignant ocular melanoma, with adjuvant treatment resulting in complete tumor remission in a canine case (Dos Santos Da Luz et al., 2023). Histological analyses of ECT-treated cutaneous melanoma metastases have revealed apoptotic changes as early as 3 h after treatment, including caspase-3 positivity, together with direct necrotic tumor damage (Bigi et al., 2016). However, comparison with UM should be interpreted cautiously given the distinct biological characteristics of the two melanoma entities and the differences in the investigated models. Currently, the exploration of ECT in UM remains in preclinical settings. Previous studies using UM cell lines, spheroids and CAM-based UM cell and PDX systems have indicated the applicability, antitumor activity and translational relevance of ECT with bleomycin using plate electrodes (Fiorentzis et al., 2018; Fiorentzis et al., 2020; Fiorentzis et al., 2019; Fiorentzis et al., 2021; Kraemer et al., 2022; Tsimpaki et al., 2024; Anastasova et al., 2024). Despite these encouraging findings, available data on UM remain limited, highlighting the need for further translational investigation.

Conventional plate electrodes limit electric field penetration and tumor coverage, particularly in larger or more compact tumor masses (Cindrič et al., 2021). To overcome these limitations, a custom bipolar electrode was designed to enable intratumoral pulse delivery while reducing the number of needle insertions required for ECT. By integrating both conductive poles within a single needle shaft, this configuration may simplify electrode placement and reduce procedural invasiveness. In contrast, currently available electrode systems rely on separate needle electrodes requiring precise spatial alignment for adequate electric field distribution. ECT is commonly performed using monopolar needle electrodes arranged in pairs (Mir et al., 2006). While this approach enables treatment of larger tumor nodules, it requires accurate electrode placement to achieve sufficient electric field coverage. The coaxial bipolar approach therefore offers several potential advantages for the treatment of UM. The simplified positioning of a single needle may be particularly relevant in the spatially restricted intraocular region, where minimizing the number of insertion sites could facilitate more targeted treatment (Merola et al., 2020; Salamanna et al., 2025). Moreover, intratumoral placement permits direct electric field delivery within the tumor tissue, which may be beneficial for compact tumor lesions that are less accessible to surface electrodes. These design characteristics distinguish the bipolar electrode from conventional plate and multi-needle arrangements and support its further development for intraocular ECT. However, direct comparative studies will be required to determine whether these features translate into improved electric field distribution and therapeutic efficacy compared with established electrode configurations.

Histological changes after treatment are consistent with the disruptive effects of ECT on tumor morphology. Structural disorganization and necrotic areas reflect EP-induced cellular damage combined with bleomycin cytotoxicity (Spiliotis et al., 2023). The more pronounced alterations observed in the *in ovo* model compared with *ex vivo* tumor samples may be attributed to differences in tumor microenvironment, including vascular integration and tissue viability within the CAM system (Ribatti, 2016; Sys et al., 2012). These factors may enhance treatment responsiveness, whereas *ex vivo* tumor fragments lack physiological support and may exhibit a more heterogeneous or attenuated response.

Reduced melanoma-associated marker expression is consistent with effective targeting of UM cells by bleomycin-based ECT. Loss of melanoma marker cocktail and Sox10 positivity likely reflects treatment-induced elimination of viable tumor cells (Alghamdi et al., 2015). Differences between models may arise from distinct biological conditions. Changes in CD31 expression suggest that ECT also affects tumor-associated vasculature within the grafts. Reduced CD31 positivity in treated *in ovo* samples indicates disruption of tumor-associated vessel structures following treatment (Sersa et al., 2008). In contrast, limited *ex vivo* changes likely reflect absence of an active vascular network. Proliferative activity remained low *in ovo*, including in untreated controls, consistent with the slow proliferative behavior described for UM (Kaliki et al., 2015). The low Ki-67 levels may additionally reflect adaptation of the transplanted tumor fragments to the CAM environment following engraftment. The higher baseline Ki-67 expression observed *ex vivo* may reflect a preserved proliferative state of the freshly excised tumor tissue, which could decline over time in the absence of physiological support.

Reversible EP alone primarily induces transient membrane permeabilization from which cells can recover, whereas the addition of bleomycin converts this process into a cytotoxic treatment by facilitating intracellular drug accumulation. Cell death marker expression suggests that ECT-induced tumor damage depends on EP parameters, bleomycin concentration and electrode design. The intratumoral bipolar electrode configuration may create local differences in electric field exposure within the tumor, resulting in variable degrees of cellular damage, as electrode geometry has been shown to influence the size and shape of the electroporated volume (Merola et al., 2020). Cells located closer to the electrode are likely subjected to higher electric field intensities and more extensive membrane disruption than in more distant tumor regions (Kranjc and Miklavčič, 2017). Together with variations in bleomycin accumulation, this may explain the non-uniform involvement of different modes of cell death. Rather than reflecting a single dominant mechanism, the observed pattern indicates heterogeneous cellular responses to combined EP-induced stress and chemotherapeutic toxicity (Peng et al., 2024). Although apoptosis, pyroptosis and necroptosis represent distinct cell death pathways, increasing evidence indicates substantial crosstalk between these processes, with their interplay depending on the cellular context (Choudhury et al., 2024; Newton et al., 2019; Fritsch et al., 2019). The treatment-associated changes in cleaved caspase-3, gasdermin D and RIP3 may therefore reflect overlapping rather than entirely independent cellular responses to ECT. The increased HMGB1 expression further supports a necrotic component of the treatment-related cell death pattern. Together with the pyroptotic and necroptotic findings, these results may suggest a potential immunogenic component of ECT-induced tumor cell death, as previously described following bleomycin-based ECT (Calvet et al., 2014). However, their downstream immune consequences cannot be fully assessed, as the CAM model possesses an incompletely developed immune system and *ex vivo* samples lack a dynamic, functional immune response. The time point of analysis, 4 days after treatment, should be considered. While early morphological and cell death-related changes following ECT have been reported within hours to days, the full cytotoxic impact of bleomycin may not yet be fully established at this stage (Bigi et al., 2016; Uršič Valentinuzzi et al., 2025). Therefore, the contribution of ECT may be partially underestimated.

These findings highlight the potential of bipolar electrode-based ECT with bleomycin in UM PDX models. Tumor disruption and evidence of multiple cell-death pathways indicate substantial antitumor activity. However, several limitations should be considered. While *in ovo* and *ex vivo* models enable the investigation of therapeutic outcomes in UM PDX, they cannot fully mimic the complex tumor microenvironment or the anatomical and physiological conditions of the human eye. *In ovo*, the CAM model provides vascular support and enables tumor engraftment and assessment of treatment responses, but its short experimental window and non-mammalian host environment limit the evaluation of longer-term treatment effects and interactions within the native ocular microenvironment (Schneider-Stock and Ribatti, 2021). In contrast, the *ex vivo* model retains the architecture and heterogeneity of the PDX tumor but lacks vascularization and physiological maintenance. Therefore, these models should be considered complementary intermediate preclinical systems for the initial investigation and optimization of treatment parameters, while further validation in appropriate animal models will be required before clinical translation. Intratumoral electrode insertion may contribute to local tissue disruption and partial destruction of tumor grafts, which could influence the observed treatment response in the experimental setting. For future intraocular applications, further refinement of electrode geometry and positioning, together with optimization of pulse delivery, will therefore be necessary to achieve reproducible tumor coverage while minimizing insertion-related trauma and unintended electric field exposure of adjacent ocular tissues. One approach could involve extraocular electrode configurations arranged along the sclera to enable targeted EP while avoiding direct tumor penetration. In addition, bipolar electrode-based ECT may have intraoperative use during endoresection of large UM. In this setting, bleomycin could be administered intravenously, with localized EP applied intraocularly to enhance intratumoral drug uptake. However, this concept requires careful evaluation of intraocular safety, drug distribution and effects on surrounding ocular tissues. ECT may also be considered as a tumor-reducing strategy in selected cases of very large UM that would otherwise require primary enucleation. A staged approach with initial tumor debulking followed by brachytherapy or teletherapy could be envisioned. Nevertheless, its feasibility and oncological safety remain to be established. Further studies are needed to assess safety and therapeutic efficacy in clinically relevant ocular tumor models.

## Data Availability

The original contributions presented in the study are included in the article/supplementary material, further inquiries can be directed to the corresponding author.
